# A new ligament-compatible patient-specific 3D-printed implant and instrumentation for total ankle arthroplasty: from biomechanical studies to clinical cases

**DOI:** 10.1186/s10195-020-00555-7

**Published:** 2020-09-02

**Authors:** C. Faldini, A. Mazzotti, C. Belvedere, G. Durastanti, A. Panciera, G. Geraci, A. Leardini

**Affiliations:** 1 1st Orthopaedic and Traumatologic Clinic, IRCCS Istituto Ortopedico Rizzoli, Via Giulio Cesare Pupilli 1, 40136 Bologna, Italy; 2 Department of Biomedical and Neuromotor Sciences, University of Bologna, 40123 Bologna, Italy; 3 Movement Analysis Laboratory, IRCCS Istituto Ortopedico Rizzoli, Via Giulio Cesare Pupilli 1, 40136 Bologna, Italy

**Keywords:** Custom-made implant, 3D-printing, Total ankle arthroplasty, Total ankle replacement, PSI, Surgical treatment

## Abstract

**Background:**

Computer navigation and patient-specific instrumentation for total ankle arthroplasty have still to demonstrate their theoretical ability to improve implant positioning and functional outcomes. The purpose of this paper is to present a new and complete total ankle arthroplasty customization process for severe posttraumatic ankle joint arthritis, consisting of patient-specific 3D-printed implant and instrumentation, starting from a ligament-compatible design.

**Case presentation:**

The new customization process was proposed in a 57-year-old male patient and involved image analysis, joint modeling, prosthesis design, patient-specific implant and instrumentation development, relevant prototyping, manufacturing, and implantation. Images obtained from a CT scan were processed for a 3D model of the ankle, and the BOX ankle prosthesis (MatOrtho, UK) geometries were customized to best fit the model. Virtual in silico, i.e., at the computer, implantation was performed to optimize positioning of these components. Corresponding patient-specific cutting guides for bone preparation were designed. The obtained models were printed in ABS by additive manufacturing for a final check. Once the planning procedure was approved, the models were sent to final state-of-the-art additive manufacturing (the metal components using cobalt-chromium-molybdenum powders, and the guides using polyamide). The custom-made prosthesis was then implanted using the cutting guides. The design, manufacturing, and implantation procedures were completed successfully and consistently, and final dimensions and location for the implant corresponded with the preoperative plan. Immediate post-op X-rays showed good implant positioning and alignment. After 4 months, clinical scores and functional abilities were excellent. Gait analysis showed satisfactory joint moment at the ankle complex and muscle activation timing within normality.

**Conclusions:**

The complete customization process for total ankle arthroplasty provided accurate and reliable implant positioning, with satisfactory short-term clinical outcomes. However, further studies are needed to confirm the potential benefits of this complete customization process.

**Level of evidence:**

5. Case report.

## Introduction

End-stage ankle joint arthritis is a disabling condition that affects about 1% of the general population, and its incidence is increasing over time, with relevant high social and economic costs [1–3]. In contrast to primary hip and knee osteoarthritis, ankle arthritis tends to be posttraumatic and typically affects younger individuals [4].

Total ankle arthroplasty (TAA) has been proposed in an effort to improve functional outcomes [4]. However, TAA outcomes have generally been unsatisfactory compared with other arthroplasties such as those at the hip and knee joints [5, 6]. For this reason, the search for successful TAA continues, leading to the development of new implants and instrumentations [7].

It has been shown in literature that a proper implant positioning is necessary for achieving good clinical results in TAA [8]. Even a small malpositioning of the implant components has a relevant impact on motion and contact pressure, which may lead ultimately to failure [9]. Great efforts have been devoted to improving surgical techniques and implant positioning, including the use of computer-aided surgery and custom cutting guides, supported also by imaging from computed tomography (CT) scans and by preoperative planning software [10]. In particular, personalized cutting guides, also known in general as patient-specific instrumentation (PSI), are customized with respect to each patient anatomy and are expected to provide more accurate component positioning [11]. However, there is still no consensus regarding which type of instrument or device is capable of providing better results [12]. In addition, most of the current customization systems are usually limited to patient-specific cutting guides and do not address personalization of implant’s dimensions and shapes. Considering the present debates in literature and taking into account the complexity of the human ankle joint, a complete custom-made system involving not only cutting guides but also prosthetic implant may represents a relevant improvement to current surgical and clinical practice for the treatment of severe posttraumatic ankle joint arthritis. As a matter of fact, most of the current TAA designs are not based on either real patient’s anatomy or physiological function, and therefore these do not seem to be able to fully reestablish gait symmetry and natural ankle motion [13].

For the above-mentioned reasons, the aim of this study is to propose a new and complete customization process for TAA, consisting of a ligament-compatible patient-specific 3D-printed implant and instrumentation. A first surgical intervention in a patient is also reported and discussed.

## Case presentation

### General information

A 57-year-old male presented at our institution in March 2019. In 2007, after a motorcycle accident, he had sustained a right articular distal tibia and fibular fractures, treated at another institute by open reduction and internal plate and screws fixation. After 7 months, nonunion was observed. Internal fixation devices were removed, and the nonunion was treated with intramedullary nailing fixation. After 2 months, infection occurred. The nail was removed, and antibiotic therapy was prescribed for several months until complete recovery.

When the patient came to our attention, he reported severe pain, loss of function, and marked limitation of ambulation. On physical examination, the right ankle presented a diffuse tenderness, with severe restriction in joint motion.

**Fig. 1:**
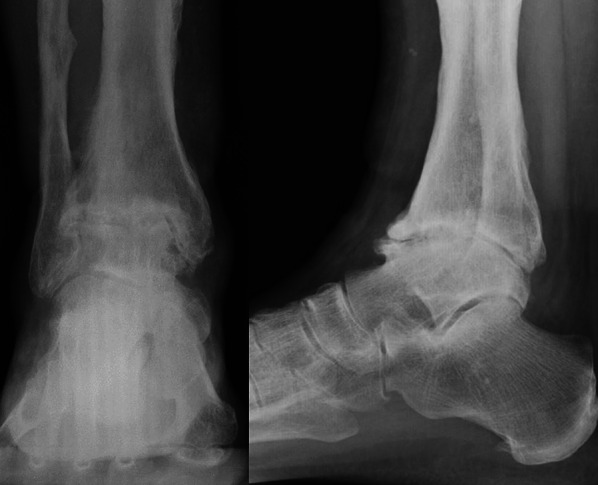
Anteroposterior and lateral weight-bearing X-rays of patient showing severe ankle arthritis

Standard radiographic examinations, including anteroposterior and lateral weight-bearing views of the ankle, showed severe posttraumatic ankle joint arthritis (Fig. 1).

Blood laboratory analyses (erythrocyte sedimentation rate, C-reactive protein, and white blood cells count) and positron emission tomography-computed tomography (PET-CT) showed no signs of infection.

The patient subjectively rated his foot and ankle pain using a 10-cm visual analog scale [14], the short-form 36-item health survey [15], and the American Orthopaedic Foot and Ankle Society ankle-hindfoot scale [16].

After considering the patient’s expectations in terms of functional outcome, a complete customization process for TAA was proposed, also considering that the dimensions of the large size of the available TAA designs were found smaller than those necessary for this ankle joint.

**Fig. 2:**
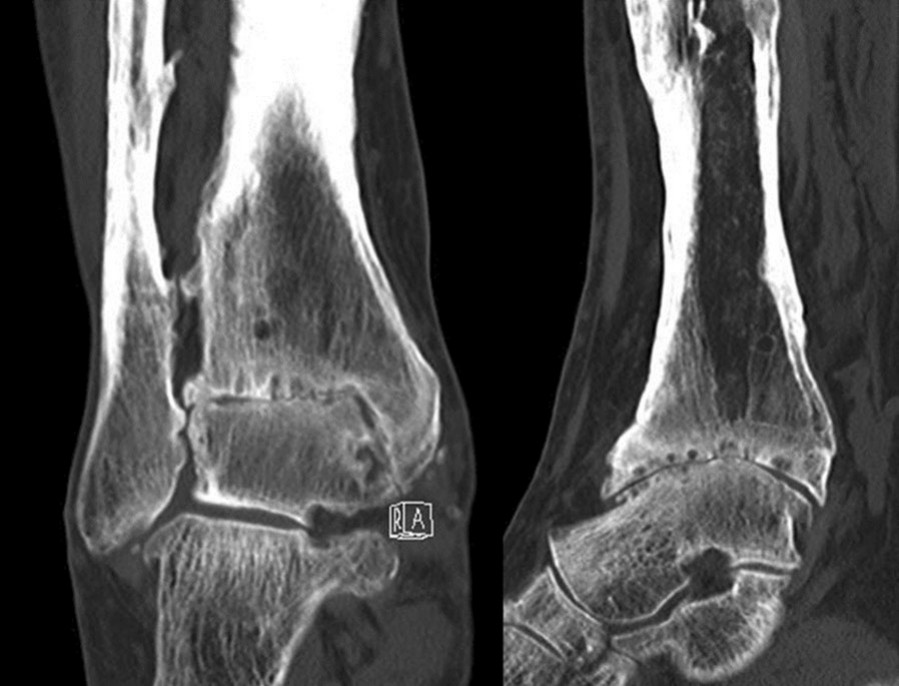
Coronal and sagittal CT sections

For this purpose, to reconstruct the patient-specific ankle morphology in three dimensions, the lower limb from mid tibia to the whole foot was scanned via CT (Fig. 2).

### Biomechanical analysis

The digital imaging and communications in medicine (DICOM) images obtained from the CT scan were processed (Mimics, Materialise, Leuven, Belgium) to obtain separate three-dimensional (3D) models of the tibia, fibula, talus, and calcaneus bones by semiautomatic segmentation of the cortical contours starting from reference Hounsfield unit values.

The three sizes of the metal components of the established BOX^®^ ankle prosthesis (MatOrtho, UK) were parameterized using Creo (PTC, MA). This entails identifying the major 3D dimensions and associating these with a current value to be modified according to the dimensions of the joint to be replaced. This would guarantee a final precise prosthesis-to-bone matching by adjusting these parameters according to the patient-specific anatomy. However, much care was taken for this process not to reshape any of the critical curves associated to the biomechanical concepts behind the design of this prosthesis, i.e., the perfect coupling between the curvatures of the tibial and talar components and the geometry of the isometric ligaments. In particular, this resulted also in the maintenance of the original meniscal inserts [17].

Using GeoMagic Control (3D Systems, SC), virtual in silico, i.e., at the computer, implantation was performed. This started by selecting the most suitable size for each metal component and adjusting to this size the parameters as mentioned above. The goal was to achieve the best possible match between the component and the bone, compatible with overall alignments and also according to surgical and clinical experience. A final compromise solution was found for this preoperative planning by collaboration between the surgeons’ supervision and the engineers. Eventually, an enlargement of the large talar component and a lengthening and posterior narrowing of the large tibial component were performed.

**Fig. 3:**
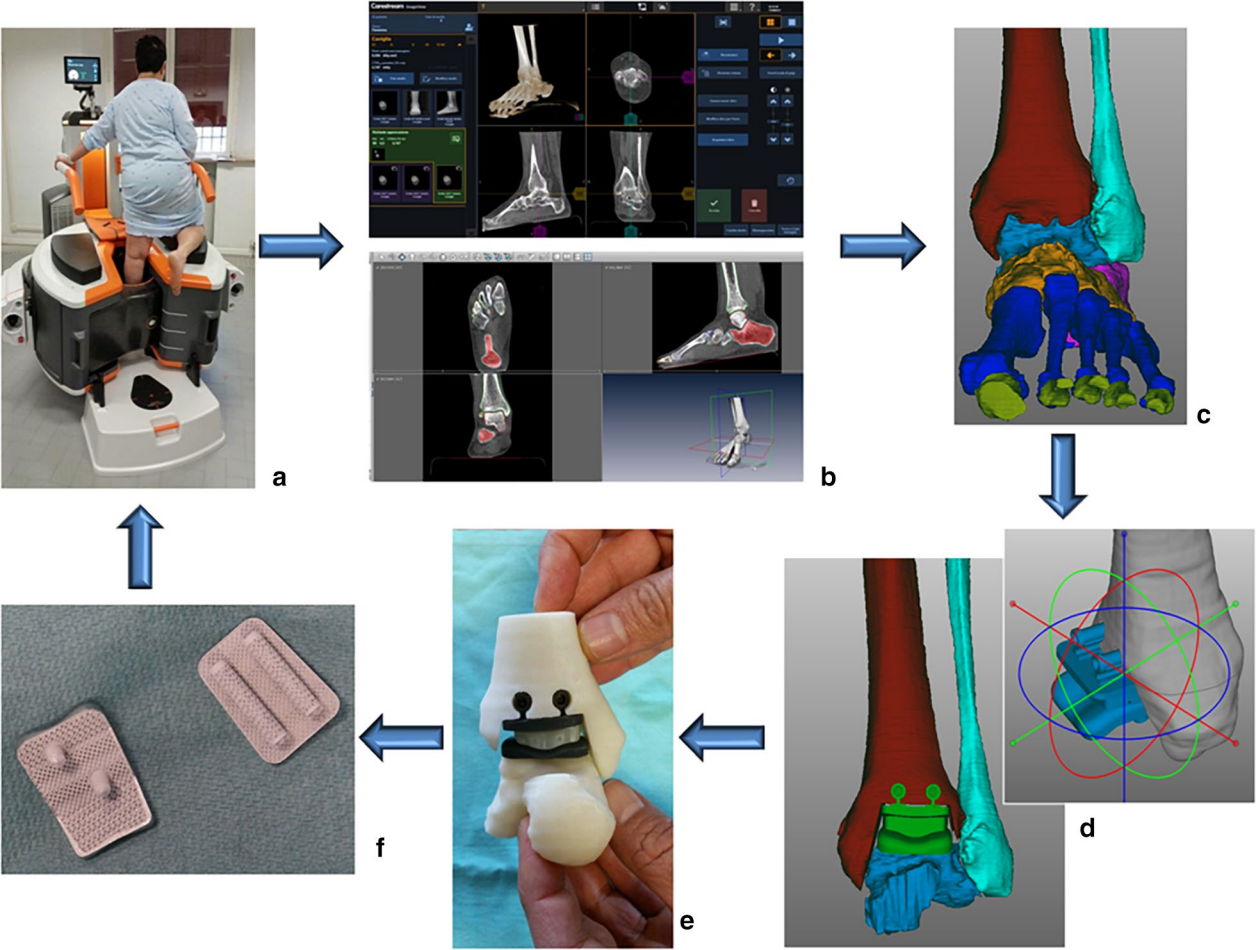
Typical flow for custom TAA design and manufacturing. **a** Typical patient during CT scan; in this case, a modern cone beam computed tomography (CBCT) device was used, with patient in weight-bearing. **b** Screenshots of medical imaging soon after scan of foot and ankle (top) and during segmentation (bottom). **c** Arthritic ankle with relevant bone models after completion of 3D reconstruction. **d** Screenshots during virtual planning and custom design: tailoring dimensions and positioning of components as well as model after final virtual implantation and corresponding bone preparation. **e** Final model of replaced ankle once 3D printed with cheap polymer powders for final check. **f** Final metal prosthesis components manufactured in Cr-Co-Mo powders just before implantation—after polishing and coating. These shall be implanted back into the original patient

**Fig. 4:**
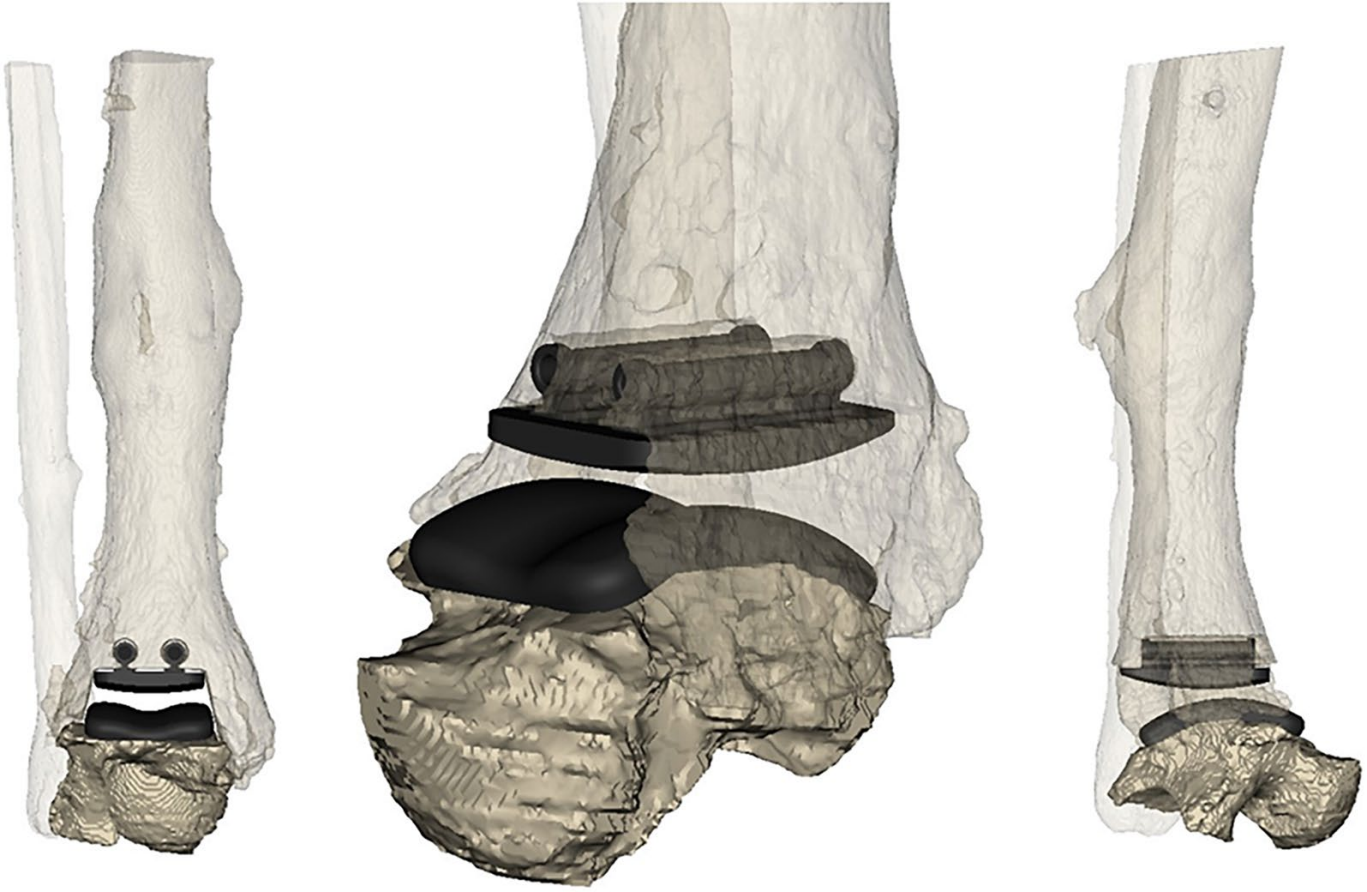
Snapshots from virtual preoperative planning after component dimensioning and positioning, and corresponding bone removal: leg in frontal (left) and lateral (right) views, and 3D view of close-up of replaced joint

Polygon manipulation (GeoMagic) was used to obtain the corresponding bone resections as well as the corresponding PSI, designed to match exactly the frontal bone of the ankle and embed all necessary guides for bone preparation, including the locations of Kirschner wires and levels of bone cuts and drills. The obtained models of the joint bones, of the custom-based prosthesis components, and of the PSI guides were printed in ABS by state-of-the-art additive manufacturing for a final check. Once the whole planning procedure was approved, the models were sent to manufacturing: the metal prosthesis components using cobalt-chromium-molybdenum powders, and the PSI using biocompatible polyamide (PA12). Before manufacturing, a porous coating interface was added to the models of the metal components for a 3D monolithic ingrowth surface, i.e., the Tri-Por^®^ and Co-Por^®^ technologies (AdlerOrtho, Milan); after manufacturing, usual polishing and hydroxyapatite coating were also performed (Figs. 3, 4).

### Surgical technique

The patient was taken to the operating room and placed in a supine position. The surgical procedure was performed with the patient under general anesthesia and using a pneumatic thigh tourniquet. The leg was sterilized up to the knee. An 8-cm anteromedial skin incision over the previous scar was performed. The reactive fibrous tissue was removed carefully to avoid bony structures resection. The cutting guides were designed to match exactly the front of the articulation in neutral position, including the osteophytes, and therefore, these were not removed during the joint surgical exposure.

**Fig. 5:**
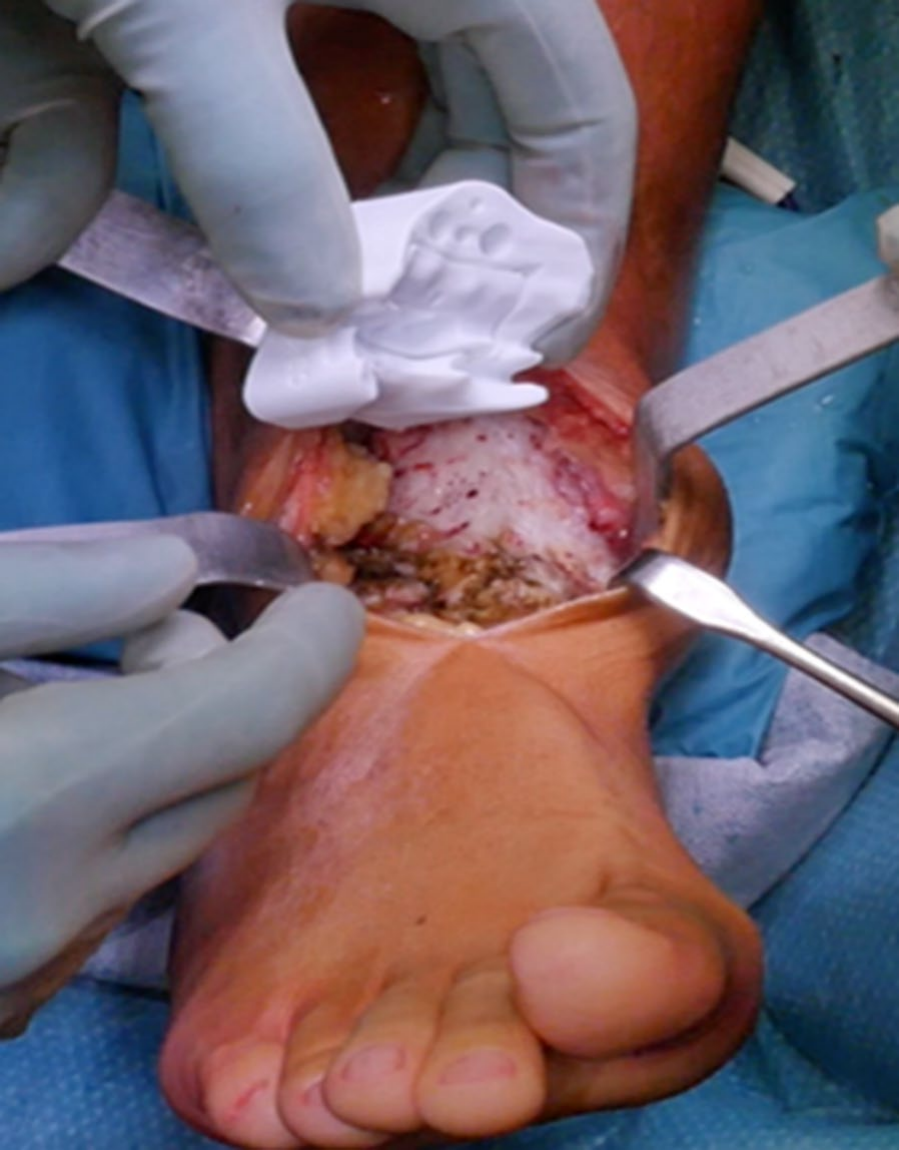
Positioning of first cutting guide designed and manufactured to match exactly the front of the articulation, including the osteophytes

**Fig. 6:**
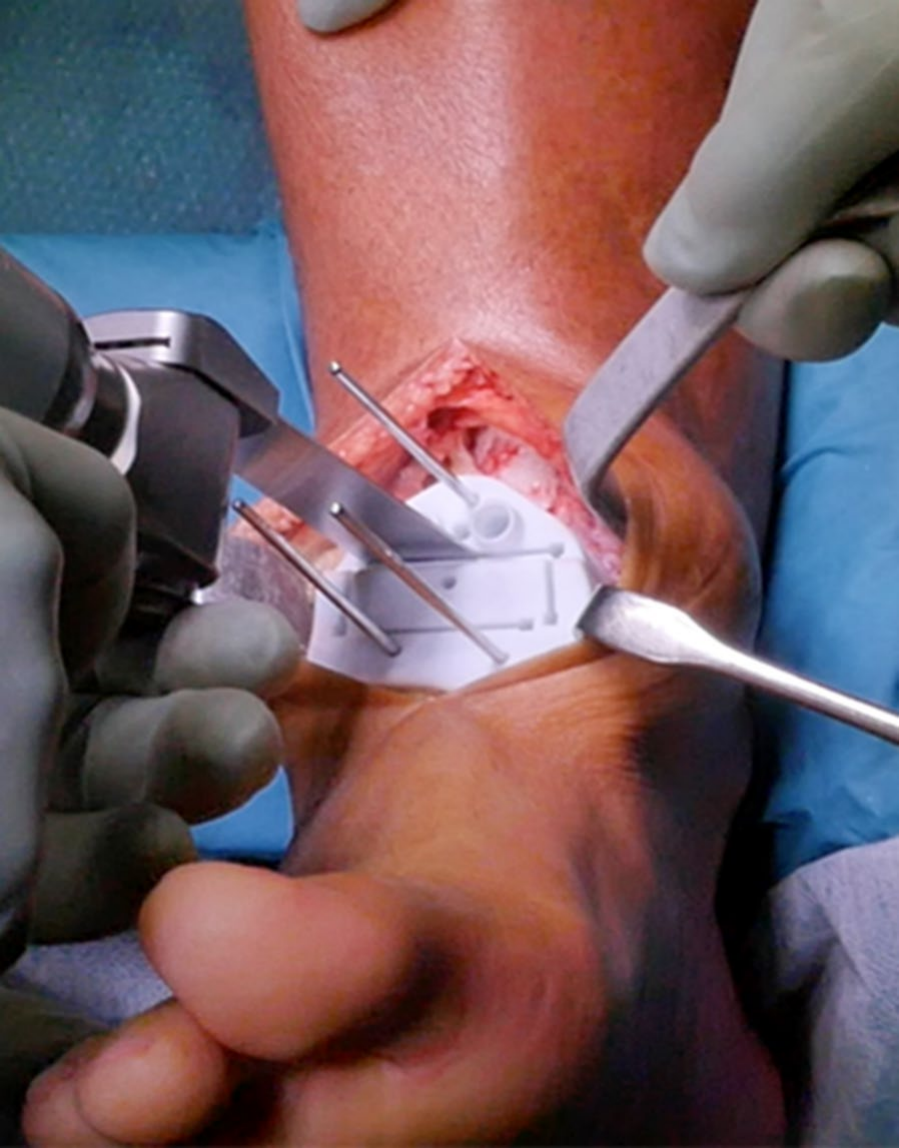
Talar and tibial resections performed using oscillating bone saw into the first cutting guide (stabilized with three Kirschner wires)

The first cutting guide was then placed over the distal tibia front surface (Fig. 5), also coming into contact with the top of the talus. Once the guide was inserted in the proper location, three Kirschner wires were placed into the dedicated holes to temporarily hold the guide into the right position. Using the oscillating bone saw, talar and tibial resections were performed (Fig. 6).

The tibial surfaces preparation was completed by drilling the two 4.5-mm-diameter holes into the guide up to the depth stop. Bone cut section and fragments were removed using a chisel. Care was taken to avoid fragments in the posterior aspect, because these may be retained by the posterior periosteum.

**Fig. 7:**
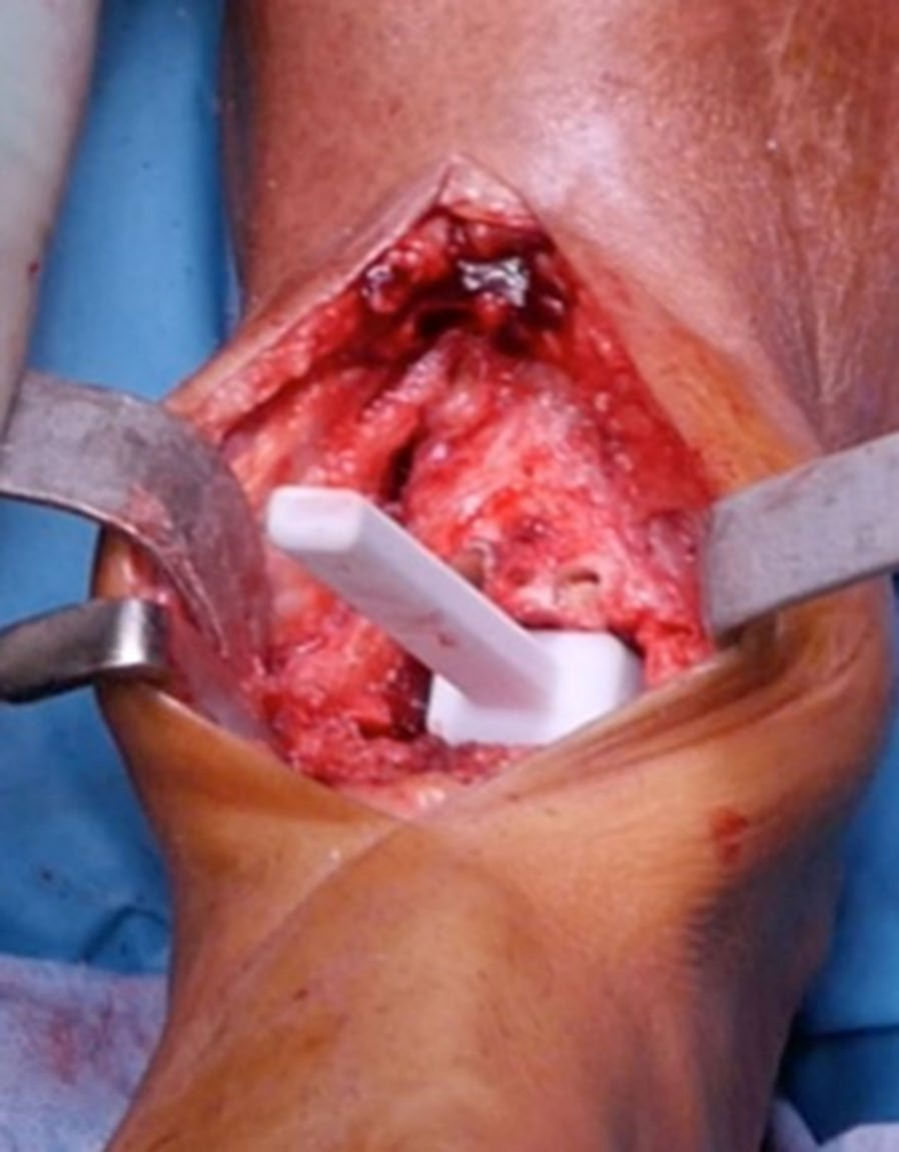
Dedicated spacer confirming proper overall amount of bone resection

A custom-made spacer was introduced to verify whether the total amount of resected bone was appropriate, i.e., this matches the aggregated thickness of the three implant components, according to the preoperative plan (Fig. 7).

**Fig. 8:**
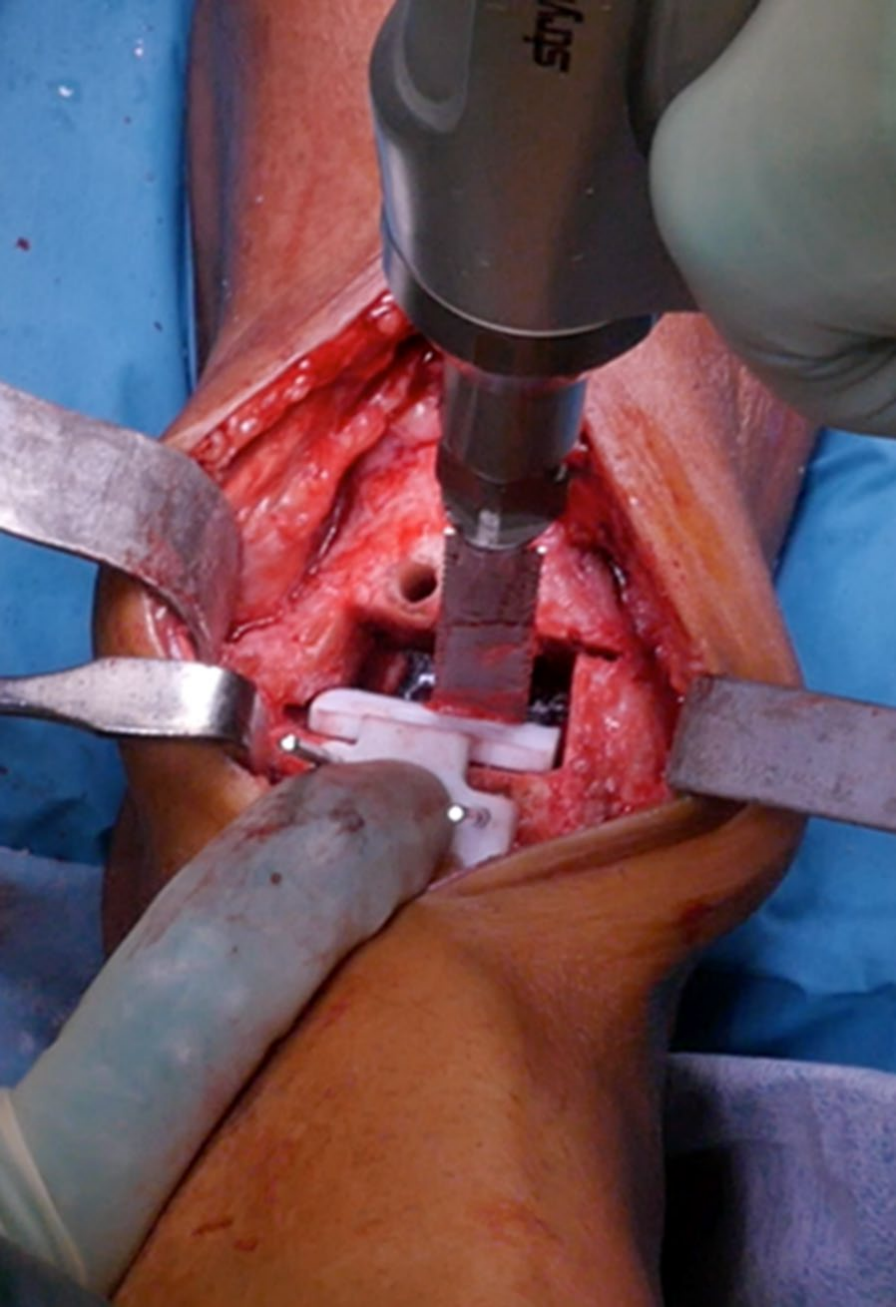
Positioning of second talar cutting guide stabilized with two Kirschner wires. Talar bone preparation for posterior chamfer and two holes for pegs completed using oscillating bone saw and drill, respectively

The foot was then placed into plantar flexion for better exposing of the talar dome. A second talar guide was inserted in the best fit location in front and above the horizontal talar cut; the two peg holes were drilled through the drill guide, and talus bone preparation was completed with the posterior chamfer cut using the oscillating bone saw (Fig. 8) and with the two holes to host the pegs of the talar component.

**Fig. 9:**
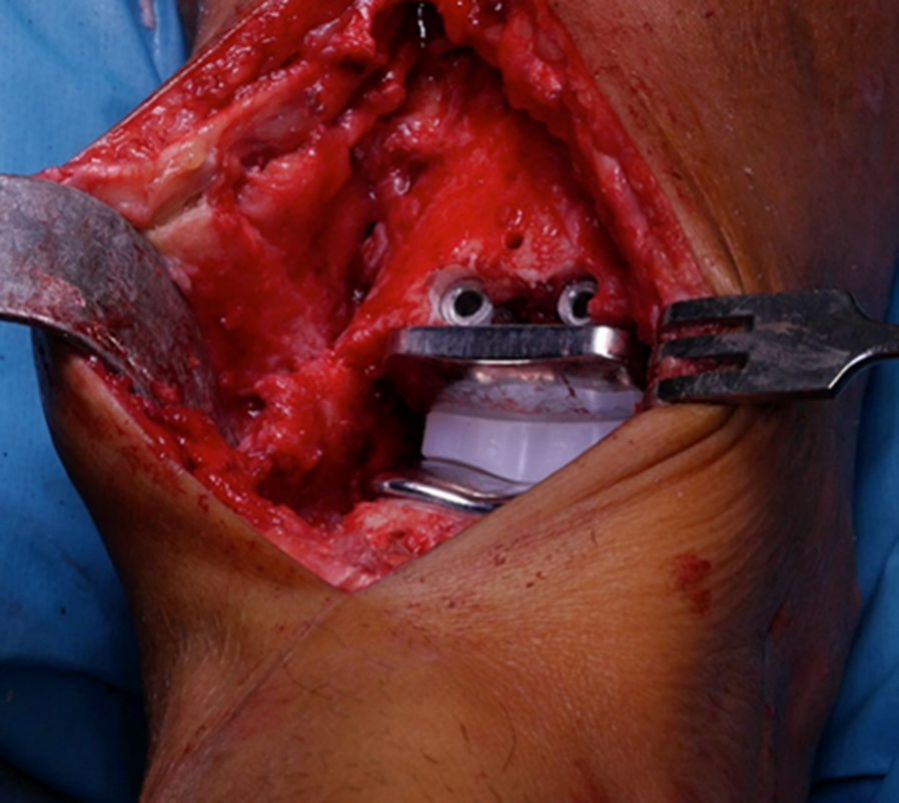
Final patient-specific 3D-printed implant in place

The cutting guide was removed and the bone section excised. Using the talar impactor, the talar final component was inserted to engage the pegs with the drilled holes. Then, the final tibial component was inserted using the tibial impactor and a spacer to avoid contact between the two superfinished metal components. The profile spacer also acted to keep the tibial component hard up against the cut bone surface during insertion, thus avoiding damage to the two holes. Trials of the meniscal bearing were performed in between the two metal components, and the final 6-mm-thick one was inserted exactly as planned (Fig. 9).

An incomplete fracture occurred at the medial malleolus during surgery, so a percutaneous screw was placed to allow early mobilization without fracture risk.

The tourniquet was released, and careful cauterization of the vessels was carried out. Suture of the anatomical planes was performed.

**Fig. 10:**
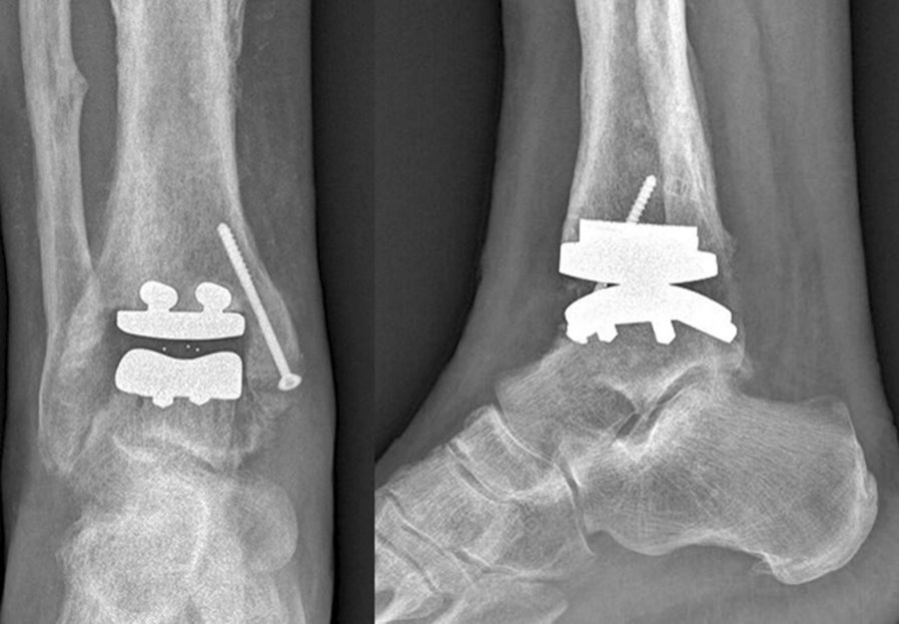
Postoperative anteroposterior and lateral X-rays

Postoperative X-rays were taken (Fig. 10).

After surgery, a non-weight-bearing plaster cast was applied below the knee. After 3 weeks, the plaster cast was removed, and a non-weight-bearing walker boot was applied for another 3 weeks. During this period, the patient underwent functional rehabilitation, including stretching exercises, water exercises, and electrical muscle stimulation. Complete and free weight-bearing was permitted 2 months after surgery.

### Results

The design, manufacturing, and implantation procedures were completed successfully and consistently; final dimensions and location for the implant corresponded to the preoperative planning.

Immediate post-op X-rays showed good implant positioning and alignment.

The intervention positively impacted on quality of life. After 4 months, the patient’s clinical abilities were restored without pain. In particular, the visual analog scale pain score improved from a preoperative score of 6.5 to 2; the short-form 36-item health survey physical component and mental component scores improved from 33.4 and 34, to 52.9 and 54 points, respectively; the American Orthopaedic Foot and Ankle Society score increased from 32 to 81 points. At this follow-up, radiographs showed the prosthesis to be stable with no signs of radiolucency around the implant. Clinically, maximum dorsiflexion and plantar flexion were measured with a goniometer and showed a range of motion of 12° and 8°, respectively.

Four months after surgery, gait analysis with state-of-the-art instruments and protocols was also performed. The kinematics and kinetics of the major joints of both lower limbs were obtained using an eight-camera stereophotogrammetric system (Vicon Motion Systems, Oxford, UK) and two force plates (Kistler, Winterthur, Switzerland), using an established protocol [18]. A quasi-physiological pattern of rotation was observed at the replaced ankle, with only limited plantar flexion at push-off, i.e., in between stance and swing phases of gait, and before landing of the foot. This, combined with normal patterns of the three components of the ground-reaction force, resulted in very limited deficits in joint moment at the ankle complex. Muscle activation timing was found within the normality, with the exception of the gastrocnemius, where a little prolonged activity, coupled with tibialis anterior, was observed.

## Discussion

The aim of this paper is to report on a recent original experience of customization for TAA. The entire procedure from medical imaging of the arthritic ankle joint to final gait analysis after replacement is described herein. This involved image analysis, joint modeling, prosthesis design, PSI development, relevant prototyping, manufacturing, and eventually implantation. For this process of developing a patient-specific implant and relevant instrumentation, which in theory could be applied to every TAA design, an own successful design was taken, which featured originally ligament-compatible shapes of the tibial and talar components. Thus the established BOX^®^ ankle based geometries [19] were selected for this customization process and for the first time investigated to minimize the critical issue of prosthesis-to-bone mismatch. Customized components were successively implanted using patient-specific cutting guides, with satisfactory early clinical and radiological results.

Despite the increasing popularity and evolution of TAA [20], results have generally been less satisfactory compared with other arthroplasties [6]. Therefore, research must continue searching for better treatments [7].

Proper implant positioning is mandatory for achieving good clinical results in TAA [8, 21, 22]. Compared with total hip and knee, the ankle joint presents a smaller surface area for load transmission and may withstand up to 500% of body weight during level walking [23]. Moreover, patients affected by ankle arthritis are usually young and active and require the implant to be strong and able to resist high-impact forces over time. Even a subtle variation in physiological joint alignment or congruency of the articulating surfaces and a slight degree of implant malpositioning can have a significant impact on joint contact pressure, which accelerates polyethylene wear, osteolysis, and loosening [9, 24].

The use of computer-aided surgery and custom-made cutting guides have been proposed in recent years to improve surgical technique, implant positioning, and theoretically clinical outcomes [10, 11]. However, there is still no clear evidence about which type of instrument or device is capable of providing better clinical results [12].

Only a few studies have reported on the potential advantages of custom-made cutting guides for TAA [10–12]. Berlet et al. [12] reported good results evaluating the repeatability of patient-specific guides’ placement and deviation between preoperative plans and actual implant placements. Hsu et al. [10] evaluated accuracy, reproducibility, and limitations in TAA components placement and alignment after using patient-specific plans and guides derived from preoperative CT scans (PROPHECY™, Wright Medical Technology, Memphis, TN). Talar implant sizing was less accurate likely due to individual surgeon preference regarding the extent of gutter debridement; however, final coronal and sagittal alignments were satisfactory. Similarly, Daigre et al. [11] reported accuracy and reproducibility of implant position with the same patient-specific guides system. Conversely, a recent study by Saito et al. [25] comparing the use of PSI with the standard referencing guide in regards to accuracy of tibial implant positioning reported similar tibial component alignment. Additionally, PSI preoperative plan reports were poor predictors of implant sizing.

Although cutting guides represent an advance in TAA, a complete custom-made system involving also prosthetic implant has not been proposed to date. The sizes of standard TAA implant components are based on statistical averages of anatomic measures. Even with a larger range of sizes, it will not be possible to address the full range of interpatient anatomical variability, in particular with regard to a high-complex articular geometry as in the arthritic ankle joint.

As a matter of fact, apart from implant sizing, most of the current TAA designs are not based on real patient’s anatomy and do not seem to be able to fully reestablish gait symmetry and natural ankle motion [13, 17]. Three-part prostheses are generally composed of an anatomic talar element, a nonanatomic flat tibial surface, and a conforming meniscus in between [26]. This combination seems unable to reproduce ligaments isometricity and, therefore, cannot restore physiological joint function, likely leading to high failure rates [17]. The BOX^®^ ankle prosthesis used as a starting point for this customization process could overcome these issues, restoring normal articular kinematics thanks to its ligament-compatible three-component design [17, 27].

A complete custom-made system seems to facilitate the surgical procedure, but surgeon experience remains an important factor, since blindly trusting the preoperative plans, customized guides, and components may lead to errors [25]. Nevertheless, the process presented herein is expected also to limit surgical errors and eventually to contribute to extension of the indication, comfort of the surgeon, and speeding of the learning curve, especially when dealing with malalignment or posttraumatic deformity requiring additional procedures.

To the best of the authors’ knowledge, this report represents the first attempt to provide a new and complete customization process for TAA, involving both cutting guides and implant. This patient-specific approach may represent a useful solution to improve current clinical practice and results, especially in young and active patients, subjecting the implant to high stress levels.

One of the main drawbacks of this technology is represented by its additional costs [28]. However, a number of relevant advantages can justify its use, such as higher successful rates after accurate preoperative planning, better implant positioning with likely smaller revisions, shorter operative time, lower fluoroscopy exposure, smaller costs for sterilization of the instrumentation, and theoretically, decreased perioperative complications.

The extent to which these potential advantages can result in considerable clinical and functional improvements compared with traditional implants remains to be studied [29]. Research must continue in the future to better investigate the effects of customization on biomechanics and bone–prosthesis stress and wear, and to consider the possibility of applying this method on a large scale, i.e., mass customization.

## Data Availability

All data generated or analyzed during this study are included in this published article.

## Abbreviations

TAA: Total ankle arthroplasty
CT: Computed tomography
PSI: Patient-specific instrumentation
